# The Effect of Incorporating Fermented Elderberries (*Sambucus nigra)* into Bread: Quality, Shelf Life, and Biological Enhancement

**DOI:** 10.3390/foods14050724

**Published:** 2025-02-20

**Authors:** Natália L. Seixas, Vanessa B. Paula, Teresa Dias, Luís G. Dias, Letícia M. Estevinho

**Affiliations:** 1 Doctoral School, University of León (ULE), Campus de Vegazana, 24007 León, Spain; natalia.seixas@ipb.pt; 2 Centro de Investigação de Montanha (CIMO), Instituto Politécnico de Bragança, Campus de Santa Apolónia, 5300-252 Bragança, Portugal; vanessapaula@ipb.pt (V.B.P.); tdias@ipb.pt (T.D.); leticia@ipb.pt (L.M.E.); 3 Laboratório para a Sustentabilidade e Tecnologia em Regiões de Montanha, Instituto Politécnico de Bragança, Campus de Santa Apolónia, 5300-253 Bragança, Portugal; 4 Instituto Politécnico de Bragança, Campus de Santa Apolónia, 5300-253 Bragança, Portugal

**Keywords:** elderberries (*Sambucus nigra)*, fermentation, bread, shelf life, biological activity

## Abstract

Elderberries, known for their antioxidant, anti-inflammatory, and antiviral properties, have traditionally been used to prevent and treat infections and boost the immune system. By increasing the quantity and quality of certain compounds, fermentation can potentially make them more effective as food additives. The aim of this study was to evaluate the effect of incorporating fermented elderberries on the bioactivity and shelf life of a traditional bread. The elderberry fermentation process was optimised using *Saccharomyces cerevisiae*, guided by a Plackett–Burman experimental design. The aim was to assess the impact of incorporating fermented elderberries into bread on its bioactive properties and shelf life. The fermentation of the elderberries was found to enhance their bioactive compounds and antioxidant activity. The total phenolic content ranged from 8.63 to 20.56 mg GAE/g (in samples without and with 2% extract, respectively). The antioxidant capacity, measured using the FRAP method, also showed a significant increase with the addition of the extract (from 9.16 to 26.66 mg Fe (II) E/g of the sample). Furthermore, bread enriched with fermented elderberry extracts demonstrated an extended shelf life during the study period.

## 1. Introduction

*Sambucus nigra*, known as elderberry, is abundant in the forests and uncultivated areas of Portugal [1,2]. Its berries have been used in traditional medicine since ancient times, and in recent decades, growing scientific evidence has highlighted their biological activities, especially their antimicrobial, antioxidant, and anti-inflammatory properties [3,4,5]. These properties are closely linked to the presence of phenolic compounds (flavonoids and phenolic acids), organic acids, anthocyanin pigments, reducing sugars, pectin, tannins, vitamins A and C, and mineral elements [6,7]. Several studies have demonstrated that adding elderberries or their extracts to foods such as wine, juice, tea, dairy, and meat products significantly improves their nutritional value and increases consumer acceptance [8]. This dual benefit makes elderberries an attractive addition to functional foods, offering health benefits alongside appealing sensory qualities.

Although some bioactive compounds are reduced during the production and processing of elderberry-based foods, they remain present in significant quantities. Key factors such as temperature, time, and pH play a crucial role in the preservation of these bioactive compounds.

Fermentation, a well-established process for creating new food products such as wine and vinegar, is gaining attention for its potential to not only extend shelf life and produce natural flavours, but also to improve the nutritional quality of foods [9]. Microorganisms involved in fermentation, whether acting individually or in combination, utilise food constituents—specifically proteins, carbohydrates, and lipids—to produce flavour and aroma compounds such as aldehydes, esters, and ketones [10]. Each microbial strain exhibits distinct aroma-forming capabilities and metabolises these substrates uniquely. As a result, fermentation can yield products with novel organoleptic properties and improved nutritional value.

Feng et al. [11] and Hang et al. [12] reported that the fermentation of elderberries with lactic acid bacteria (LAB) led to an increase in total phenolic compounds and total amino acids, suggesting that fermentation with LAB enhances both the nutritional value and bioactive properties of elderberries.

Bread is one of the most widely consumed foods worldwide and plays a vital role in a balanced diet due to its high nutritional value, being rich in complex carbohydrates (primarily starch), fibre, B-complex vitamins, and minerals [13]. According to Portuguese legislation, bread is defined as the product obtained through the kneading, fermentation, and baking of wheat, rye, triticale, or maize flour, either individually or in combination. It may also include salt and other ingredients, such as additives [14,15]. Additionally, bread serves as an excellent medium for delivering functional ingredients to consumers in a familiar and widely accepted form. Due to their beneficial properties, the inclusion of natural additives improves the nutritional profile of bread and can also have a positive impact on human health [16,17].

The main objective of this study was to optimise the fermentation process of elderberries by *Saccharomyces cerevisiae* using a Plackett–Burman experimental design. The aim was also to assess the effect of incorporating fermented elderberries on the bioactivity and shelf life of a traditional bread loaf.

## 2. Materials and Methods

### 2.1. Sampling

In this study, the dehydrated elderberries were supplied by the InovTerra Association, located in Vila Pouca de Salzedas, Portugal, in 2021. The dehydrated sample contained some impurities from the tree and handling processes, which were manually removed prior to use. Before homogenisation, the elderberries were frozen at −24 °C and were subsequently milled into a fine powder using an IKA Tube Mill (Staufen, Germany).

### 2.2. Reagents

The reagents magnesium sulphate (MgSO_4_), monopotassium phosphate (KH_2_PO_4_), urea, and sodium hydroxide (NaOH) were purchased from Merck KGaA (Darmstadt, Germany). The Rose Bengal agar culture medium was supplied by Liofilchem (Roseto degli Abruzzi, Italy). The modified iron sulphite agar culture medium, Baird-Parker (BP) culture medium, and *Bacillus cereus* Selective Agar (MYP) culture medium were purchased from HiMedia Laboratories (Modautal, Germany). The SimPlate kit for counting total coliforms/*Escherichia coli* and the 1–2 Test Kit for *Salmonella* were supplied by Biocontrol^®^ (Bellevue, WA, USA). Plate Count Agar (PCA) medium was provided by Panreac (AppliChem, Barcelona, Spain). Ethanol, 99.8% pure, was obtained from Carlo Erba Reagents (Chaussee du Vexin, France). The reagents Folin–Ciocalteau, 2,2-diphenyl-1-picrylhydrazyl (DPPH•), aluminium chloride, sodium carbonate, quercetin, gallic acid, Trolox, Ferric-Reducing Antioxidant Power (FRAP), acetic acid, 2,4,6-tri(2-pyridyl)-s-triazine (TPTZ), iron(III) chloride (FeCl_3_), iron(II) sulphate heptahydrate (FeSO_4_.7H_2_O), hydrochloric acid (HCl), and other reagents used in our study were purchased from Sigma Chemical Co. (St. Louis, MO, USA). Petroleum ether was purchased from Chem-LAB (Zedelgem, Belgium). Potassium dihydrogen phosphate (KH_2_SO_4_) and selenium (Se) were supplied by Scharlau (Barcelona, Spain).

### 2.3. Optimisation of Fermentation Conditions

The optimisation of fermentation conditions for elderberry was performed using the Plackett–Burman design with 15 experiments (Table 1), involving 6 parameters at 3 levels each, as follows: urea (1.00, 1.50, and 2.00 g/L), MgSO_4_ (0.50, 1.00, and 1.50 g/L), KH_2_PO_4_ (0.50, 1.00, and 1.50 g/L), and pH of the culture medium (4.50, 4.75, and 5.00); inoculum concentration of *Saccharomyces cerevisiae* was tested at 10^5^, 10^6^, and 10^7^ colony forming units (CFU/mL); amounts of elderberries (50.0, 150.0, and 250.0 g), with a final volume of 250 mL.

The fermentation tests were conducted in 500 and 1000 mL Erlenmeyer flasks. The salts required for the different experimental conditions were accurately weighed and added to each flask. Then, 150 mL of distilled water was added to dissolve the salts, and the initial pH of the medium was adjusted. The solution’s volumes were adjusted to 250 mL and were sterilised by autoclaving at 121 °C for 15 min. After sterilisation, the flasks were allowed to cool and then the appropriate amount of sample was added to each flask, according to the experimental design. The flasks containing the samples were then pasteurised in a water bath at 65 °C for 15 min. After cooling on ice, the inoculum (*Saccharomyces cerevisae*) was added. The Erlenmeyer flasks were incubated at 25 °C in an orbital incubator (Incubated Shaker SIF6000R). Fermentations was monitored daily for 144 h by evaluating the following parameters: flask weight, pH, and colony forming units (CFU).

### 2.4. Preparation of Fermented Elderberry Extract

For the preparation of the fermented elderberry extract (FEE), the optimal fermentation process was selected based on the experimental design described above. The selected conditions were 1.0 g/L urea; 0.5 g/L MgSO_4_; 0.5 g/L KH_2_PO_4_; 50 g sample; and pH 4.5. The ideal inoculum concentration was determined to be 10^5^ CFU/mL, based on the experimental design. At the end of the fermentation, the product was frozen at −18 °C and lyophilized (−60 °C under vacuum) for later hydroethanolic extraction. The extraction was performed using a hydroethanolic solution (40:60, *v*/*v*) at pH 0.9, which had been optimised for the extraction of total phenolic compounds from elderberries. A total of 3.16 g of the lyophilised sample was weighed, and 50 mL of hydroethanolic solution was added. The mixture was stirred overnight and then filtered. After filtration, the extract was concentrated on a rotary evaporator (using a water bath at 40 °C and rotating the flask at 200 rpm) (model IKA V8). 

### 2.5. Bread Production

Bread was prepared using type 65 flour (40 g)—this type of flour is a commercial, moderately refined flour that is ideal for baking, with a medium ash content, a good elasticity, and a balanced flavour—baker’s fresh yeast (probiotic microorganism) (0.3 g), salt (Portuguese “Vatel” salt) (0.10 g), water (30 mL), and different concentrations of FEE (0.0%, 0.5%, and 2.0%) to produce loaves of approximately 50 g each. Bread production started with the mixing of the following ingredients: flour, water, yeast, and salt (the yeast was hydrated in warm water). The dough was worked (kneaded) for 10 to 15 min until it reached a smooth and elastic texture. Then, it was left for the first fermentation, resting for 1 h until it doubled in size.

After the initial fermentation, the dough was shaped into the desired form and underwent a second fermentation (30 min). The bread was then baked in an oven at 250 °C for approximately one hour. Once baked, the bread was left to cool and stored at room temperature in a dry environment. A total of six loaves were prepared: two without FEE (control), two with 0.5%, and two with 2.0% FEE. The bread was stored in paper bags purchased from a supermarket to simulate typical consumer storage conditions. The samples were subjected to analysis immediately after production (T0) and two days later (T2) for microbiological, chemical, and physicochemical analyses. 

### 2.6. Microbiological Analysis

The purpose of the microbiological analyses was to evaluate the quality of the product obtained from the fermented elderberries (at 0 h and after 144 h) and the bread samples (at time 0 and after 2 days). 

The quantification of moulds and yeasts was performed using DRBC (Rose Bengal) agar medium. Growth was observed from the second day of incubation at 25 °C for yeasts and up to the fifth day of incubation for moulds [18].

Sulphite-reducing clostridial spores were analysed and quantified by incorporation after the inactivation of the sample. The tubes were placed in anaerobic conditions and incubated at 37 °C for 5 days [19].

The SimPlate kit (Biocontrol) was used to test and quantify total coliforms and *Escherichia coli*, following the manufacturer’s recommendations [20].

Total coliforms were assessed by observing the colour change in the wells of the KIT plate from blue to pink, while the detection and quantification of *E. coli* were performed by counting fluorescing wells under ultraviolet (UV) light at 365 nm. The results were interpreted using the conversion table provided by the manufacturer.

*Staphylococcus aureus* enumeration was performed with Baird-Parker medium (BP-HIMEDIA), supplemented with egg yolk, and the spread technique was used. Incubation was performed for 48 h at 37 °C [21]. After this period, characteristic colonies (grey colonies with a transparent halo) and non-characteristic colonies were selected to test for the presence of positive coagulase. These were inoculated into Brain Heart Infusion (BHI) broth and incubated at 37 °C for 24 h. Rabbit plasma was then added and incubated for 12 h. *S. aureus* colonies were identified if clot formation was observed at the bottom of the tube.

*Salmonella* spp. were tested using the 1–2 TEST kit from BioControl and Ambifood (Porto, Portugal) [22]. After the pre-enrichment and enrichment of the samples at 37 °C, the kits were prepared according to the method recommended by the manufacturer. The kits were incubated at 37 °C for 24 h, after which the presence or absence of an immunoprecipitation band was observed, indicating positivity for *Salmonella.*

*Bacillus* cereus was quantified by plating 0.1 mL of each dilution onto Petri dishes containing selective agar for *B. cereus* (MYP) [23]. Plates were incubated at 30 °C for 24 to 48 h. Positive colonies were identified by their pink colour.

The culture medium used to count total mesophilic aerobic bacteria was Plate Count Agar (PCA), and the incorporation technique was used. Plates were incubated at 30 °C for 48 to 72 h [24].

In general, microbial counts were expressed in colony forming units (CFU) per gram or millilitre of sample.

### 2.7. Bioactive Compounds and Antioxidant Activity

Bioactive compounds and antioxidant activity were analysed for both the fermented berries and the bread enriched with the extract. The elderberry extract was also analysed for these compounds.

To carry out these analyses, hydroethanolic extracts were prepared from the elderberry, the fermented product, and the bread by weighing 1 g of each freeze-dried product, adding 25 mL of the hydroethanolic solution, stirring for one hour, filtering, and evaporating. The extract obtained was then dissolved in 10 mL of the ethanolic solution.

The method reported by Singleton et al. [25], with modifications, was used for the determination of total phenolic compounds (TPCs). For this analysis, 0.5 mL of sample, 2.5 mL of Folin–Ciocalteu reagent (10%), and 2 mL of 75 g/L sodium carbonate were pipetted. The solution was left in the dark for 2 h and its absorbance was read at 760 nm (Spectrophotometer UV-3100PC, VWR International LLC, Avantor, Radnor, PA, USA). The results were calculated from a calibration curve using gallic acid (GA) as a standard and with concentrations ranging from 4 to 60 mg/L, expressed in mg GAE per g of sample.

The flavonoid content (TFC) was determined using the method described by Woisky and Salatino [26] with modifications. For this analysis, 2.5 mL of sample and 2.5 mL of 2% (*w*/*v*) AlCl_3_ were pipetted. The solution obtained was allowed to rest in the dark for 1 h. After this period, the absorbance was measured at 420 nm. Quercetin (Q) was used as a standard to construct the calibration curve using concentrations between 0.48 and 3 mg/L. The results were expressed in mg QE per g of sample.

Antioxidant capacity was assessed using the DPPH• and FRAP methods.

- DPPH• (2,2-diphenyl-1-picrylhydrazyl)

This method was performed as described by Hatano et al. [27] with slight modifications. For this test, 0.3 mL of sample and 2.7 mL of DPPH• reagent (2.0 × 10^−4^ M) were pipetted. The solutions were kept in the dark for 60 min and the absorbance was measured at 517 nm. The results were then expressed in mg TroloxE/g of sample, using a calibration curve with concentration ranges between 12.6 and 100.8 mg/L, established using Trolox.

- FRAP (Ferric-Reducing Antioxidant Power)

Antioxidant activity was also determined using the FRAP method according to Berker et al. [28]. FRAP reagent was prepared by mixing 0.3 M acetate buffer (pH 3.6), 10 mM TPTZ, and 20 mM FeCl_3_ in a 10:1:1 ratio. For the analysis, 0.1 mL of sample was pipetted, followed by the addition of 3 mL of FRAP reagent and 0.3 mL of deionized water. The solutions were incubated in the dark for 6 min, and absorbance was measured at 595 nm. The results were expressed in mg of Fe(II)E per gram of sample, using a calibration curve constructed with ferrous sulphate heptahydrate at concentrations between 25 and 350 mg/L.

### 2.8. Physicochemical Properties

The physicochemical composition of the bread samples was assessed by determining the following parameters: moisture, ash content, protein content, fat content, and pH. Energy and carbohydrate content were also calculated.

The moisture content was determined by AOAC 925.10 [29], measuring weight loss in an oven at 105 °C until a constant weight was achieved. The moisture percentage was calculated as the mass difference between the initial weight and the weight after drying.

The ash content was determined according to AOAC 923.03 [30] via the incineration of the samples in a muffle furnace at 600 °C (BOX Furnace—model 51894). The quantification of the mineral residue was calculated as the difference between the sample´s mass and the ash´s mass. The pH of the bread samples was evaluated according to [31], using a potentiometer (Mettler Toledo model, Mumbai, India) with a combined pH electrode. The method recommended by AOAC 920.85 [32] was used to quantify the total fat content of the bread samples. This method recommends Soxhlet extraction (Behrotest, Labor-Technik, Düsseldorf, Germany) using petroleum ether as solvent. Approximately 2 g of the sample was placed in a cartridge, which was placed in a previously weighed flat-bottomed flask containing 170 mL of light petroleum, and the system was heated for 6 h. After extraction, the solvent was distilled off and the residue was transferred to an oven at 105 °C for one hour. After cooling, the flask was weighed. To determine the total protein content of bread, we used AOAC 950.36 [33]. Two grams of the homogenised sample was weighed and placed in a Kjedahl tube. A volume of 12 mL of concentrated sulfuric acid, along with two catalyst tablets (composed of K_2_SO_4_ and Se), was added. The tube was placed in the mineralisation unit (DK 8-Heating Digester, Italy) at 420 °C for 60 min. After cooling the samples to 50 °C, distillation was started in the UDK Semi-Automatic Distillation Unit, followed by titration with 0.2 mol/L HCl. This method makes it possible to quantify the protein content of the samples analysed. To determine the percentage of protein in the bread samples, the percentage of free nitrogen present in the samples is first calculated and then the percentage of protein is calculated using the following formula: % protein = % nitrogen × 5.7.

### 2.9. Statistical Analysis

All data treatments were performed using the statistics software R 4.4.0 GUI 1.80 Big Sur Intel build (The R Foundation for Statistical Computing, Vienna, Austria), a free software environment for statistical computing and graphics. The statistical analysis included mean comparisons using Student’s *t*-test, one-way ANOVA, two-way ANOVA, and the Generalised Linear Model (GLM). The data were tested for normality and homogeneity of variance assumptions using the Shapiro–Wilk test on residuals and Levene’s Test, respectively. When the assumption of variance homogeneity was violated, the validity of the ANOVA results was compromised. Therefore, the GLM approach was applied to confirm whether there were significant differences in the means of the response variable. Taking into account the interactive terms and the factors under study, this offers a robust alternative due to its flexibility and ability to handle heteroscedasticity. GLMs extend traditional linear models by allowing the specification of a link function that relates the mean of the response variable to the linear predictors (an identity link function was used), as well as a variance structure that accommodates non-constant variance, enabling the model to adapt to the characteristics of the data. To determine which group means are statistically different, pairwise comparisons were performed using Tukey’s Honest Significant Difference (HSD) test.

## 3. Results and Discussions

### 3.1. Optimising Fermentation Conditions

**Figure 1:**
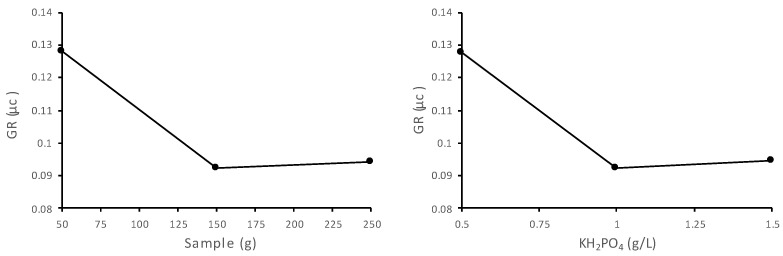
Influence of the variables that contributed to the growth rate (GR).

For optimising the fermentation conditions for elderberry, 15 different experiments were carried out, varying six parameters (X1: inoculum concentration; X2: urea; X3: MgSO_4_; X4: KH_2_PO_4_; X5; pH; and X6: sample quantity). Three concentration levels were used. It was found that variables X4 and X6 were the most significant contributors to yeast growth, suggesting that they should be selected for further tests. An analysis of Figure 1 shows that growth was optimal in the presence of 0.5 g/L KH_2_PO_4_ and 50 g of sample.

In other words, the maximum specific growth rate (0.128 µC) was obtained at the lowest concentration of KH_2_PO_4_ tested for a sample quantity of 50 g. In this context, all the fermentations in our study were carried out under the following conditions: 1.0 g/L urea; 0.5 g/L MgSO_4_; 0.5 g/L KH_2_PO_4_; 50 g sample; and pH 4.50. The optimal inoculum concentration, determined based on experimental design analysis, was found to be 10^5^ CFU/mL.

### 3.2. Microbial and Chemical Profile of the Fermented Elderberries (FEs)

#### 3.2.1. Microbiological Analysis of the Fermented Elderberries

From the microbiological analyses (yeast and moulds, total coliforms/*E. coli*, *S. aureus*, *Clostridium*, *Salmonella*, and *B. cereus*) of the fermented elderberries, only yeast growth was detected (5.53 and 4.44 CFU/mL for T0 and T144, respectively). Analysis of the data showed statistically significant differences (*p* < 0.001) in the growth of moulds and yeasts between the two time points. The decrease in yeast population after 144 h of elderberry fermentation may be due to several factors, such as nutrient depletion, ethanol accumulation, pH changes, or competition with other microorganisms that contribute to the decrease in viable yeast cells.

#### 3.2.2. Chemical Analysis of the Fermented Elderberries

The average results obtained for the elderberry extract (EE) and the fermentations are summarised in Table 2. All tests were performed in triplicate. Analyses were performed at time zero (T0) and after 144 h (T144).

Table 2 shows that all the evaluated parameters exhibited a twofold increase, both with the addition of the fermentation medium components and from T0 to T144. Fermentation enhanced the antioxidant properties with DPPH• and FRAP, by 2.64 and 2.08 times. This increase is likely attributed to the production or biotransformation of phenolic compounds and flavonoids by the yeast. Furthermore, the bioactive compounds, as indicated by total phenolic content (TPC) and total flavonoid content (TFC), showed a twofold increase, with values rising by 2.24 and 2.04 times, respectively, during the fermentation process.

In fermented products, Kwaw et al. [34] also observed an increase in the content of anthocyanins, phenolic compounds, flavonoids, and antimicrobial activity, which corroborates with the results obtained in this work. The increase in bioactive compounds and biological properties in the fermentation medium (T0) compared to the elderberry extract may be related to the reagents incorporated in the medium, i.e., urea, KH_2_PO_4_, and MgSO_4_. KH_2_PO_4_ is a buffer that influences the action of the enzymes that carry out the reactions, such as hydroxylation or methylation, responsible for the degradation of the various compounds present in the fermentation medium. The antioxidant capacity of anthocyanins is also directly influenced by pH [35].

The ANOVA results regarding the TPC, TFC, and antioxidant capacity (DPPH• and FRAP) values showed significant differences (*p*-values < 0.001) between the values obtained in the three matrices analysed (letters a, b, and c).

The increase in total phenolic compounds can be attributed to the action of the yeast, suggesting that not only does it release these compounds from the plant material, but it also modifies them during its cellular metabolism.

According to YEO et al. [36], the yeast *S. cerevisiae* produces secondary metabolites during fermentation (aldehydes, esters, acids, phenols, biomass, and methanol, among others), which, in many situations, are responsible for different biological activities such as of an antimicrobial, antifungal, or antiviral nature. Also, according to the study conducted by Sabel et al. [37], yeasts, especially those of the genus *Saccharomyces*, were more resistant to the phenolic compounds tested compared to lactic and acetic bacteria, suggesting that yeasts are more resistant to the action of phenolic compounds.

### 3.3. Microbial and Chemical Profile of Bread

**Figure 2:**
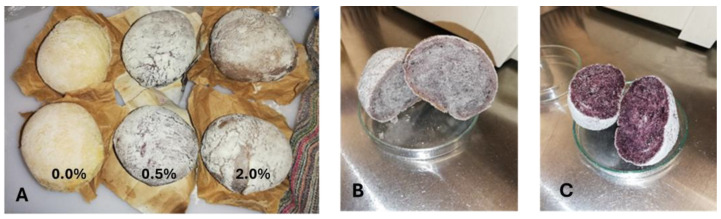
Photos of bread made with (**A**) 0.0%, 0.5%, and 2.0% of fermented elderberry extract; (**B**) 0.5% of fermented elderberry extract; and (**C**) 2.0% of fermented elderberry extract.

Figure 2 shows the three breads prepared with different concentrations of fermented elderberry extract (FEE: 0.0%, 0.5%, and 2.0%), highlighting their distinct colours.

#### 3.3.1. Microbiological Analysis of Bread

All the microorganisms studied (mould and yeast, total coliforms/*E. coli*, *S. aureus*, *Clostridium*, and total mesophilic) were absent in all the samples analysed, as expected due to the high baking temperature. After 2 days of storage, only the total mesophilic microorganisms were quantified; there was a decrease as a result of the increase in the concentration of FEE added to the bread (0.0%—5.18 ± 0.14 log CFU/mL; 0.5%—4.93 ± 0.03 log CFU/mL; 2.0%—3.87 ± 0.04 log CFU/mL). The number of aerobic mesophiles in the samples without added extract was significantly different from that in the bread with 2% extract (*p* = 0.001). According to Silva et al. [38], the number of aerobic mesophilic microorganisms is an indicator of the quality of products, raw materials, and handling conditions, i.e., low levels indicate the good general hygiene of a product; in this context, the samples studied can be considered satisfactory and do not represent increased risks to consumer health [39,40,41,42]. These results suggest that elderberries could be used to extend the shelf life of bread, as their ability to ensure microbiological safety was superior to that of bread containing only conventional ingredients.

#### 3.3.2. Chemical Analysis of Bread

**Figure 3:**
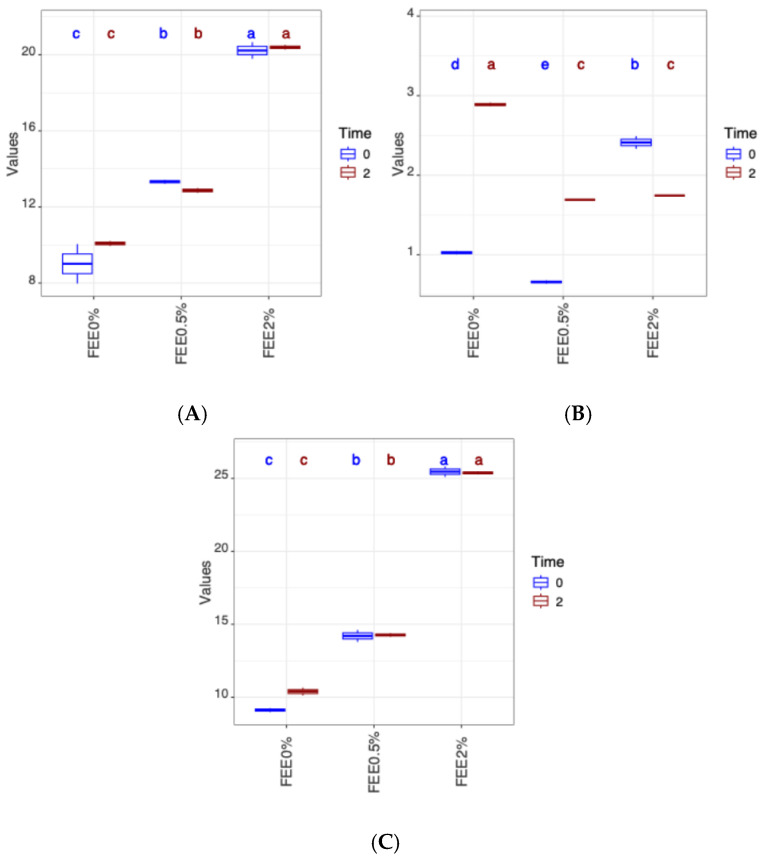
Two-way ANOVA with box plots for bread with and without fermented elderberry extract, measured at 0 and 2 days of storage. (**A**) TPC, (**B**) TFC. and (**C**) FRAP results. TPC: total phenolic content; TFC: total flavonoids content; FRAP: Ferric-Reducing Antioxidant Power. In each variable, different letters indicate significant mean differences (a–e).

Table 3 shows the average values obtained for total phenolics and flavonoids, as well as antioxidant activity (DPPH and FRAP methods), for the three types of bread produced, in addition to the results of the two-way ANOVA study. Figure 3 presents the boxplots of the two-way ANOVA data of the TPC, TFC, DPPH, and FRAP results.

From analysing Table 3, it was observed that there were no statistically significant differences in the quantification of TPC from T0 to T2 in all the samples studied, which means that they remained stable during the shelf life of the bread. The same can be observed for the antioxidant capacity determined using the FRAP method. It can also be seen that the extract factor (0.0%, 0.5%, and 2.0% FEE) had a significant effect on all the parameters studied (*p* < 0.001). There were significant differences, both between T0 and T2 and with the amount of extract added (*p* < 0.001), for the antioxidant capacity determined using TFC.

Karakaya et al. [43] studied the behaviour of anthocyanins (pigments belonging to the flavonoid group) over time in bakery products (bread, baguette bread, and biscuits) enriched with this compound and found that baking and storage (room temperature) did not affect the stability of anthocyanins, while maintaining their bioactive properties. This confirms the results obtained in this study relating to the behaviour of total phenolics.

Figure 3A shows that the amount of phenols increased as the FEE content added to the bread increased. However, in the samples where FEE was incorporated, the content of these compounds remained practically constant throughout the storage time. The phenol content of the control was the lowest, increasing from time 0 to the final time. Analysing the two times simultaneously for the same situation, it was found that in the bread incorporated with extracts, the phenolic content increased with the amount of extract added and remained practically constant during storage.

Analysing Figure 3B, for the two times analysed, the sample with 2.0% FEE had the highest total flavonoid content (2.36 ± 0.12 and 2.90 ± 0.03 mg eq Q/g sample, respectively), while the bread sample with 0.5% FEE had the lowest value (0.65 ± 0.02 mg eq Q/g sample). There were no significant differences between the samples with added FEE on the second day of storage. These results show that increasing the concentration of FEE added to the bread did not increase the total flavonoid content at T2.

The results shown in Figure 3C indicate that on the day the bread was made (T0), the highest value of antioxidant capacity was obtained in the control sample (247.23 ± 0.94 mg TroloxE/g sample), with this formulation also having the highest value at the end of the storage process. In the bread samples with FEE added, an increase in antioxidant activity was observed as a function of the incorporated extract content. For all the bread samples analysed, there was an increase in antioxidant activity from T0 to T2, as assessed using the DPPH method, with the sample containing 0.5% FEE showing the greatest increase.

The results obtained in the different bread samples at T0 and T2 for the antioxidant capacity assessed using the FRAP method (Figure 3C showed an increase due to the addition of FEE to the bread samples. Comparing the two times studied and the three samples, in the control sample (0.0% FEE) there was a slight increase in antioxidant activity from T0 to T2, while this variation was practically zero in the other two formulations.

The increase in antioxidant capacity assessed using the FRAP method may be related to the TPC of the bread samples, since the behaviour of these two parameters (Figure 3A,C) was similar [17]. 

#### 3.3.3. Physicochemical Analysis

Table 4 summarises the results obtained for the parameters of protein, fat, moisture, ash, pH, carbohydrates, and energy for the bread samples at the two times studied (T0 and T2 days).

Table 4 shows that, overall, the results obtained in the three samples for the different parameters studied decreased from T0 to T2. In the case of the control sample (0.0% FEE), protein and pH stand out, with significant differences between the two times studied, whereas in the sample with 0.5% FEE, all the parameters decreased except moisture, which remained constant over time. The 2.0% FEE sample only showed significant differences in the protein and fat parameters. The values obtained for protein content (between 7.93 ± 0.29 and 8.44 ± 0.06%) are within the range recommended by the General Directorate of Health [44] for white bread (8.40%).

The fat content of the samples studied was low (values ranging from 0.122 ± 0.003 to 0.030 ± 0.002 %) compared to that described by Dr. Ricardo Jorge at the National Health Institute [45], who indicates that the value for fat in white bread is 2.20%. These results can be explained by the fact that no type of fat (olive oil, oil, or butter) was added to the bread during its preparation.

The obtained moisture values ranged from 31.75 ± 0.21 to 35.22 ± 0.59 %, and there was no trend in relation to the addition of FEE and storage time (T0 and T2) for the different samples evaluated. Martini et al. [46] also obtained average moisture values ranging from 26.28 to 33.87 % for hot dog buns.

The ash content ranged from 1.01 ± 0.17 to 2.32 ± 0.15%. The values of this parameter in the sample with 2.0% FEE at T2 were almost double those observed in the control at T0, showing statistically significant differences between these two samples. The values obtained for ash in this study, ranging from 1.16 to 2.04 %, were identical to those reported by Martini et al. [46] in French bread, hot dog bread, and biscuits. According to Coutinho et al. [47], the ash content of samples is used to assess the purity and authenticity of foods, as it reflects the amount of inorganic minerals present in their composition.

Harbers and Nielsen [48] also reported that ash content is made up of inorganic substances of nutritional importance, i.e., the higher its content in food, the greater its contribution to health. In this context, the bread sample with the highest percentage of elderberry extract (2.0% FEE) was the one with the highest percentage of ash, which means that the consumption of bread with the highest amount of extract will have a greater contribution to human health.

The pH values obtained for the three samples at the two times studied ranged from 4.12 ± 0.07 to 5.57 ± 0.05. These values decreased from T0 to T2, as well as with the increase in the percentage of FEE incorporated into the bread. These results may be related to the increase in the amount of anthocyanins (the most abundant phenolic compound in elderberries) in the bread samples, to which a higher concentration of FEE was added. Bratu et al. [49] showed that in dried berry infusions, the greater the presence of anthocyanins in the samples, the lower the pH.

The total carbohydrate content of the different bread samples studied ranged from 85.99 to 87.21 g/100 g of bread. These values are slightly higher than those stipulated by the Directorate General for Health for bread known as ‘white bread’ or ‘wheat bread’ (57.3 g/100 g). These quantities may be related to the lower fat content obtained in this work.

The energy values obtained (377.47 and 381.93 Kcal/100 g) were also higher than those established by the Directorate General for Health (289 Kcal/100 g bread). This study also showed that the energy value of bread decreased slightly, although the differences were not significant (*p* = 0.0638), as the percentage of FEE added to the samples increased, suggesting that bread with a higher amount of extract can be considered more beneficial for human health.

Regarding fat, since the values obtained were very small and some of the errors were too high, it was considered more appropriate not to carry out a statistical analysis for this parameter.

Considering the overall results of the project, it can be concluded that elderberry holds significant potential as a functional ingredient in fermented products. However, further studies are essential to gain a better understanding of its variability and to expand upon the knowledge of its impact on various food applications. Assessing the variability of elderberries across Portugal is crucial for evaluating the feasibility of standardising the extract, including its chemical profile and biological properties. Additionally, selecting suitable probiotic microorganisms for the fermentation process and optimising the extract’s characteristics are critical steps to enhance its application in the food industry.

We are not aware of any studies relating to the incorporation of elderberries in traditional bread. There are also no data in the literature on the optimisation of the fermentation process with a probiotic yeast, or on the effect of using a fermented product on the characteristics of the bread, particularly its preservation and bioactivity.

## 4. Conclusions

Elderberries are rich in phenolic compounds and flavonoids, with potential for use as a functional food. Fermentation with *S. cerevisiae* showed that lower amounts of sample and KH_2_PO_4_ improved the process, increasing total phenolic compounds and antioxidant activity. After pasteurisation, the fermented product showed a low microbial load, making it bioactively interesting for incorporation into bread.

When fermented berries (FEE) were added to the bread, there was an increase in total phenolic compounds, antioxidant activity (especially via the FRAP method), and an improvement in conservation, with a reduction in the count of mesophilic aerobic microorganisms. At the same time, there were physicochemical changes, such as an increase in ash and a decrease in pH.

The results suggest that fermented elderberries show great potential for enhancing bread by improving its bioactive properties, enriching its nutritional value, and extending its shelf life.

However, future research should evaluate the sensory aspect and toxicity of this product.

## Figures and Tables

**Table 1:** Experimental design to optimise fermentation conditions for elderberries.

Assay	Inoculum (CFU/mL)	Urea (g/L)	MgSO_4_ (g/L)	KH_2_PO_4_ (g/L)	pH	Sample (g)
1	10^6^	1.50	1.00	1.00	4.75	150
2	10^7^	2.00	1.50	0.50	4.50	50
3	10^5^	2.00	0.50	1.50	5.00	50
4	10^7^	1.00	0.50	0.50	5.00	50
5	10^7^	2.00	0.50	1.50	5.00	250
6	10^5^	2.00	1.50	0.50	5.00	250
7	10^5^	1.00	1.50	0.50	5.00	250
8	10^6^	1.50	1.00	1.00	4.75	150
9	10^7^	1.00	1.50	1.50	4.50	250
10	10^7^	2.00	0.50	0.50	4.50	250
11	10^5^	1.00	0.50	1.50	4.50	250
12	10^7^	1.00	1.50	1.50	5.00	50
13	10^5^	1.00	0.50	0.50	4.50	50
14	10^5^	2.00	1.50	1.50	4.50	50
15	10^6^	1.50	1.00	1.00	4.75	150

**Table 2:** Results of the chemical analysis of the fermented product and application of ANOVA.

	EE	T0	T144
TPC (mg GAE/g S)	40.80 ± 0.21 ^c^	79.63 ± 4.39 ^b^	178.30 ± 1.78 ^a^
TFC (mg QE/g S)	1.27 ± 0.05 ^c^	13.66 ± 0.69 ^b^	27.87 ± 0.34 ^a^
DPPH• (mg TroloxE/g S)	21.69 ± 0.08 ^c^	62.90 ± 1.09 ^b^	116.05 ± 1.89 ^a^
FRAP (mg. Fe (II)E/g S)	139.81 ± 1.29 ^c^	204.14 ± 7.60 ^b^	425.44 ± 4.88 ^a^

Results expressed in mean of analysis ± standard deviation. EE: elderberry extract; TPC: total phenolic content; TFC: total flavonoids content; DPPH•: 2,2-Diphenyl-1-picrylhydrazyl; FRAP: Ferric-Reducing Antioxidant Power; S: sample; GAE: gallic acid equivalent; QE: quercetin equivalent; TroloxE: Trolox equivalent; Fe (II)E: ferrous ion equivalent. In each variable, different letters indicate significant mean differences (a–c).

**Table 3:** Results of the chemical analysis of the bread with and without fermented elderberry extract, measured at 0 and 2 days of storage.

Bread	Time (Days)	TPC (mg eq. GAE/g S)	TFC (mg eq. QE/g S)	FRAP (mg Fe (II)E/g S)
0.0% FEE	0	8.63 ± 1.23 ^c^	1.02 ± 0.03 ^d^	9.16 ± 0.16 ^c^
	2	10.25 ± 0.33 ^c^	2.90 ± 0.03 ^a^	10.45 ± 0.28 ^c^
0.5% FEE	0	13.08 ± 0.44 ^b^	0.65 ± 0.02 ^e^	14.30 ± 0.44 ^b^
	2	13.22 ± 0.62 ^b^	1.72 ± 0.05 ^c^	14.16 ± 0.22 ^b^
2.0% FEE	0	20.56 ± 0.72 ^a^	2.36 ± 0.12 ^b^	25.61 ± 0.45 ^a^
	2	20.40 ± 0.12 ^a^	1.77 ± 0.05 ^c^	25.66 ± 0.51 ^a^

Results expressed in mean of analysis ± standard deviation. FEE: fermented elderberry extract; TPC: total phenolic content; TFC: total flavonoids content; DPPH: 2,2-Diphenyl-1-picrylhydrazyl; FRAP: Ferric-Reducing Antioxidant Power; S: sample; GAE: gallic acid equivalent; QE: quercetin equivalent; TroloxE: Trolox equivalent; Fe (II)E: ferrous ion equivalent. In each variable, different letters indicate significant mean differences (a–e).

**Table 4:** Results of the physicochemical analysis of the bread with and without fermented elderberry extract, measured at 0 and 2 days of storage.

Bread	Time (Days)	Protein (%)	Moisture (%)	Ash (%)	pH	CH (g/100 g)	EV (Kcal/100 g)
0.0% FEE	0	8.44 ± 0.06 ^a^	33.24 ± 1.13 ^a^	1.01 ± 0.17 ^b^	5.57 ± 0.05 ^a^	86.97 ^a^	381.93 ^a^
	2	8.33 ± 0.03 ^ab^	33.74 ± 0.26 ^a^	1.50 ± 0.11 ^b^	5.39 ± 0.01 ^ab^	86.33 ^a^	379.73 ^a^
0.5% FEE	0	8.27 ± 0.05 ^ab^	32.28 ± 0.62 ^a^	1.32 ± 0.38 ^b^	5.30 ± 0.06 ^bc^	86.76 ^a^	380.68 ^a^
	2	7.93 ± 0.29 ^b^	34.16 ± 0.14 ^a^	1.24 ± 0.06 ^b^	5.11 ± 0.04 ^c^	87.21 ^a^	380.85 ^a^
2.0% FEE	0	8.17 ± 0.05 ^ab^	3.22 ± 0.59 ^a^	2.07 ± 0.74 ^a^	4.21 ± 0.08 ^d^	86.02 ^a^	377.84 ^a^
	2	8.31 ± 0.00 ^ab^	31.75 ± 0.21 ^a^	2.32 ± 0.15 ^a^	4.12 ± 0.07 ^d^	85.99 ^a^	377.47 ^a^

Results expressed in means of analysis ± standard deviation. FEE: fermented elderberry extract; CH: carbohydrates; EV: energy value. In each variable, different letters indicate significant mean differences (a–e).

## Data Availability

The original contributions presented in this study are included in the article. Further inquiries can be directed to the corresponding author.
